# *Candidatus* Rickettsia colombianensi in ticks from reptiles in Córdoba, Colombia

**DOI:** 10.14202/vetworld.2020.1764-1770

**Published:** 2020-09-03

**Authors:** Jorge Miranda, Lina Violet-Lozano, Samia Barrera, Salim Mattar, Santiago Monsalve-Buriticá, Juan Rodas, Verónica Contreras

**Affiliations:** 1 University of Córdoba, Institute of Tropical Biology Research, Córdoba, Colombia; 2 Corporación Universitaria Lasallista, Colombia; 3 University of Antioquia, Colombia, Colombia

**Keywords:** arthropod vectors, reptile trade, tick-borne diseases, wild animals

## Abstract

**Background and Aim:**

Wildlife animals are reservoirs of a large number of microorganisms pathogenic to humans, and ticks could be responsible for the transmission of these pathogens. *Rickettsia* spp. are the most prevalent pathogens found in ticks. This study was conducted to detect *Rickettsia* spp. in ticks collected from free-living and illegally trafficked reptiles from the Department of Córdoba, Colombia.

**Materials and Methods:**

During the period from October 2011 to July 2014, ticks belonging to the family Ixodidae were collected, preserved in 96% ethanol, identified using taxonomic keys, and pooled (between 1 and 14 ticks) according to sex, stage, host, and collected place for subsequent DNA extraction. *Rickettsia* detection was performed using real-time polymerase chain reaction (RT-PCR), followed by conventional PCR to amplify a larger fragment of the *gltA* and *16S rRNA* genes. The amplicons were sequenced using the Sanger method, and the nucleotide sequences were subjected to BLAST analysis to identify homologous sequences in GenBank, after which phylogenetic analysis was performed using the MEGA X software.

**Results:**

In total, 21 specimens of nine species of reptiles were sampled, from which 805 *Amblyomma dissimile* ticks were collected, but only 180 ticks were selected to create 34 groups. The DNA of *Rickettsia* spp. was detected in 30/34 (88%) groups. The sequences of the gene *gltA* and *16S rRNA* revealed a 100% identity with *Candidatus* Rickettsia colombianensi (GenBank: KF905456 and GenBank: KF691750).

**Conclusion:**

*A. dissimile* was the only tick found in all the sampled reptiles. The presence of *Candidatus* Rickettsia colombianensi in reptile ticks could represent a public health problem due to the risk of transmission to humans and the introduction of microorganisms to other geographical areas.

## Introduction

Wildlife animals are reservoirs of a large number of microorganisms that are pathogenic to humans. Approximately 61% of human diseases are known to have a zoonotic origin, and 75% of emerging zoonoses worldwide are associated with wild animals [1]. Vertebrate mammals and animals belonging to other taxa, including reptiles and amphibians, serve as hosts for blood-sucking ectoparasites such as ticks [2]. Ticks are the second vector, after mosquitoes, responsible for the significant transmission of vector-borne diseases [3], and the vertebrate hosts can be infected by various protozoa, bacteria, and viruses, which cause 17% of infectious diseases worldwide [3-5].

An increasing number of studies suggest that reptiles are competent hosts of microorganisms such as *Rickettsia* spp., *Borrelia* spp., and *Ehrlichia* spp. [6-8]. Colombia, with abundant biodiversity, has high rates of illegal trade in wild animals, covering both domestic and international markets [9]. Animals that are trafficked illegally often carry ticks with them, thereby representing a route of dispersal and introduction of tick species and pathogens to new geographical areas [10-12].

The bacteria belonging to the genus *Rickettsia* are a type of *Alphaproteobacteria*, obligate intracellular, possessing Gram-negative characteristics but are best visualized by Gimenez staining [13]. *Rickettsia* spp. comprise one of the most common microorganisms detected in reptile ticks [6], and to date, 23 *Rickettsia* species and several other *Candidatus* are known to be associated with at least 18 tick species found in 42 species of amphibian and reptile hosts reported in 36 countries [8]. *Rickettsia* has been found in the tissues of lizards, which could represent a model for studies on the epidemiology and pathogenesis [6,14].

*Candidatus* Rickettsia colombianensi has been identified in reptiles from Mexico, Honduras, Colombia, and Brazil, and it is probable that its distribution is related to the presence of *Amblyomma dissimile*, which ranges from the north of Mexico to the Southern Cone of America [8]. Some researchers consider that *Candidatus* Rickettsia colombianensi has an endosymbiont relationship with reptile ticks [8]. However, it has also been reported that it has a remarkable cytopathic effect on Vero cells [15], and its gene sequences relate it among the Rickettsia spotted fever group, which causes diseases in humans [15,16].

Regarding the potential role of reptiles as reservoirs, there is a scarcity of information, and the issue requires further evaluation [14]. Therefore, the aim of this study was to detect *Rickettsia* spp. in ticks collected from free-living and illegally trafficked reptiles, which were seized by authorities in the Department of Córdoba (Colombia).

## Materials and Methods

### Ethical approval

The Ethics Committee of the Institute of Tropical Biology Research at the University of Córdoba approved the ethics protocol to obtain the samples of ticks from animals. The study incorporated management procedures to preserve the integrity of the animals according to the resolution 8430 of the Ministry of Health of Colombia.

### Type of study and geographical area

From October 2011 to July 2014, we carried out a descriptive study in some areas of Department of Córdoba. The ticks samples were taken for convenience due to the availability of the reptiles specimens coming from seizures of illegal traffic and collections from biological studies. The region of the study is a highly fragmented tropical dry forest, with vegetation modified by human agricultural activities such as livestock and corn, rice, vegetables, citrus, and other tropical fruits. The average annual temperature is 29°C, reaching 38.5°C in the dry season, and 27°C in the rainy season.

**Figure-1:**
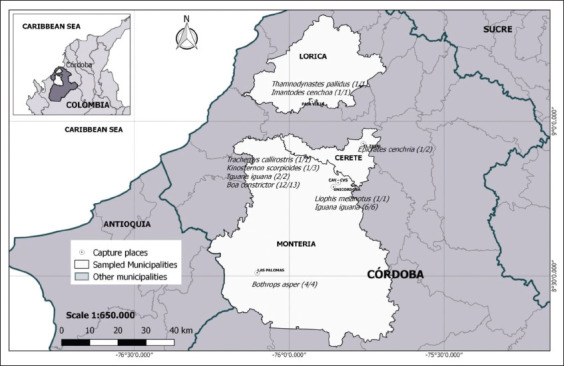
Area and sites of specimen collection, Cordoba, Colombia [Source: This map was designed by the authors of the manuscript and Misael Oviedo and Yulisa Velasquez executed the idea using QGIS (VERSION 3.14) software].

Ticks from illegally trade reptiles were collected by the environmental authority of Colombia in charge of wildlife (CAV-CVS) located in the city of Montería, Department of Córdoba (8°48’04.0’’ N - 75°50’75,9’’ W). Besides, free-living reptile ticks collected in the municipality of Montería, at the University of Córdoba (08º45’15.71” N-75º51’28.01” W) and “*Las Palomas*” (08°30’37.1” N-76°06’12.9” W); “*El Zapal*,” municipality of Cereté (8°55’40.80” N-75°45’40.93” W), and “*Paja Vieja*,” municipality of Lorica (09°03’50.7” N-75° 55’34.4” W) (Figure-1).

#### Tick collection sites and taxonomic identification

Ticks belonging the family Ixodidae were collected in all stages directly from individuals of the species *Boa constrictor* (common boa), *Iguana iguana* (green iguana), *Kinosternon scorpioides* (scorpion mud turtle), *Bothrops asper* (fer-de-lance viper), *Erythrolamprus melanotus* (Shaw’s dark ground snake), *Epicrates maurus* (rainbow boa), *Thamnodynastes gambotensis* (snake), *Imantodes cenchoa* (blunt-headed tree Snake), and *Trachemys callirostris* (the Colombian slider turtle). The ticks collected from the reptiles were preserved in 96% ethanol and then transported to the Institute of Tropical Biology Research of the University of Córdoba. The identification of ticks was performed using the dichotomous taxonomic keys described by Barros-Battesti [17,18]. Ticks were grouped according to host, sex, stage, and geographic area, comprising 1-14 adults, 4-9 nymphs, and 1-7 larvae.

### Molecular detection of *Rickettsia* spp.

The tick pools were placed in tubes containing 200 μl PBS. DNA extraction was performed using the QIAamp Mini DNA kit (QIAGEN, CA, USA), according to the manufacturer’s instructions. For the detection of *Rickettsia* spp., real-time polymerase chain reaction (RT-PCR) was performed using the primers CS-5: GAGAGAAAATTATATATCCAAATGTTGAT and CS-6 AGGGTCTTCGTGCATTTCTT and a hydrolysis probe -FAM-CATTGTGCCATCCAGCCTACGGT-BHQ-1 for the detection of a fragment of citrate synthase gene (*gltA*, 147 bp) as described by Labruna *et al*. with some modifications [19]. Two negative controls were included in each test. DNA of *Rickettsia amblyommatis* was used as a positive control, and internal control of phage lambda genomic control DNA (TIB MOLBIOL, NJ USA) was also used in each amplification reaction. Next, the samples determined to be positive by RT-PCR were further analyzed by conventional PCR using two sets of primers, the CS-78 GCAAGTATCGGTGAGGATGTAAT and CS-323 GCTTCCTTAAAATTCAATAAATCAGGAT for detecting a larger fragment of the *gltA* gene of 401 bp. For better identification of the rickettsia species, a fragment of 426 bp of the 16 rRNA gene was also amplified with the primers fD1 AGAGTTTGATCCTGGCTCAG and Rc16S. 452n AACGTCATTATCTTCCTTGC [19,20]. For each reaction, 5 μl of molecular grade water was included as a negative control and 5 μl of *R. amblyommatis* DNA was used as a positive control. The PCR products were visualized by electrophoresis on a 1.5% agarose gel. These products were purified for subsequent sequencing using a QuickLink^™^ gel extraction kit (Invitrogen) according to the manufacturer’s instructions. The prevalence of infection caused by *Rickettsia* spp. in ticks was expressed as the minimum infection rate (MIR), which is the minimum detectable percentage of ticks infected by *Rickettsia* in a group. This was based on the fact that each PCR-positive group contained at least one infected tick [16].

### Phylogenetic analysis

Sequencing was performed using the Sanger method. Subsequently, the obtained DNA partial sequences were aligned and compared with other sequences of *Rickettsia* available in GenBank. The identification of species was conducted by sequence homology using the MEGA X software (https://www.megasoftware.net/), in which multiple alignments of the sequences reported for the genus *Rickettsia* were made available in GenBank. For constructing the phylogenetic trees, the distances between homologous sequences were calculated using Kimura’s two-parameter model. For each gene analyzed, a phylogenetic tree was constructed using the maximum likelihood method. The confidence values for the individual branches of the resulting tree were determined by bootstrap analysis with 1000 repetitions [21].

## Results

### Identification of ticks

**Figure-2:**
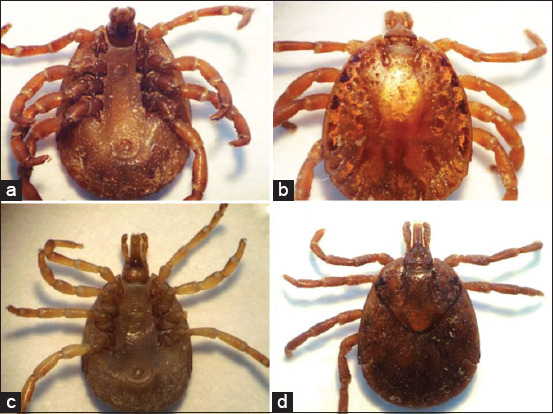
Adult specimen of *Amblyomma dissimile*: (a) Male frontal; (b) male dorsal; (c) frontal female; and (d) dorsal female view.

From a total of 21 reptiles, 805 ticks of *A. dissimile* were identified (Figure-2). According to their development stage, 499 (61.9%) were nymphs, 173 (21.5%) were males, 96 (11.9%) were females, and 37 (4.6%) were larvae. Based on the host, 653 (81%) ticks were collected from eight snakes (*B. constrictor*), and 68 (8.4%) ticks were collected from one snake (*E. maurus*). The other hosts and ticks collected in this study and the sites of origin are described in Table-1.

**Table-1:** Hosts, number, and stage of development of ticks collected and pools analyzed by RT-PCR.

Host species		Collected ticks					Pools of ticks analyzed by RT-PCR
	
Species	Origen	F	M	N	L	Total	
*Trachemys callirostris*	CAV-CVS* (Montería)	1	-	-	-	1	(1F)
*Kinosternon scorpioides*		2	2	-	-	4	(2M) (2F)
*Kinosternon scorpioides*		-	3	4	-	7	(3M)
*Boa constrictor*		6	14	-	-	20	(14M) (4N)
*Boa constrictor*		-	-	367	-	367	(7N)
*Boa constrictor*		10	32	52	-	94	(12M) (5F)
*Boa constrictor*		9	24	-	-	33	(6M) (4F)
*Boa constrictor*		-	3	-	-	3	(3M)
*Boa constrictor*		44	52	-	-	96	(7F) (7M)
*Boa constrictor*		10	14	7	-	31	(7F) (7M)
*Boa constrictor*		4	5	-	-	9	(5M)
*Iguana iguana*		2	4	-	-	6	(2F) (4M)
*Iguana iguana*	Unicordoba (Montería)	-	-	-	8	8	(8L)
*Iguana iguana*		2	6	-	7	15	(2F) (6M) (7L)
*Iguana iguana*		-	4	-	-	4	(4M)
*Iguana iguana*		-	1	-	-	1	(1M)
*Erythrolamprus melanotus*		-	-	-	14	14	(14L)
*Bothrops asper*	Las Palomas (Montería)	2	1	9	7	19	(2F) (1M) (9N) (7L)
*Thamnodynastes gambotensis*	Paja Vieja (Lorica)	-	-	-	1	1	(1L)
*Imantodes cenchoa*		-	-	4	-	4	(4N)
*Epicrates maurus*	El Zapal (Cereté)	4	8	56	-	68	(5N) (7N)
Total		96	173	499	37	805	34 (180)

* CAV-CVS=Centre for animal care of wildlife of Cordoba; UNICORDOBA (University of Córdoba). F=Females, M=Males, N=Nymphs, L=Larvae, -=0, RT-PCR=Real-time polymerase chain reaction

### Molecular detection

Of the 805 ticks sampled, 180 were used to set up 34 groups. The DNA from *Rickettsia* spp. was detected in 30/34 (88.23%) groups of ticks analyzed by real-time PCR. Regarding the groups of ticks, according to reptile host species, we detected an infection prevalence range of 33-100% (Table-2).

**Table-2:** Host and percentage of infected groups with *Rickettsia* spp.

Number of specimens	Host species	Collected ticks (%)	Analyzed pools by PCR/positive pools (%)	Minimum infection rate* (%)
1	*Trachemys callirostris*	1 (0.12)	1/1 (100)	1/1 (100)
1	*Thamnodynastes gambotensis*	1 (0.12)	1/1 (100)	1/1 (100)
1	*Imantodes cenchoa*	4 (0.50)	1/1 (100)	1/4 (25)
1	*Erythrolamprus melanotus*	14 (1.74)	1/1 (100)	1/14 (7)
1	*Bothrops asper*	19 (2.36)	4/4 (100)	4/19 (21)
1	*Epicrates maurus*	68 (8.45)	2/1 (50)	1/12 (8)
2	*Kinosternon scorpioides*	11 (1.37)	3/1 (33)	1/7 (14)
5	*Iguana iguana*	34 (4.22)	8/8 (100)	8/34 (23)
8	*Boa constrictor*	653 (81.12)	13**/12 (92)	12/88 (13)
21	Total	805 (100)	34/30 (88)	30/180 (16)

* Minimum infection rate: Represents the minimum percentage of ticks infected by *Rickettsia* in a group, this is based on the fact that each positive PCR group contains at least one infected tick.

** 88 ticks were taken to make up the 13 groups. PCR=Polymerase chain reaction

Through conventional PCR, the *gltA* gene (401 bp) and the *16S rRNA* gene were amplified in 18 (60%) of the 30 positive pools by real-time PCR; six groups corresponded to the ticks of individuals of *I. iguana*, four pools were from *B. asper*, five pools were from *B. constrictor*, and one pool was from each of the following: *E. melanotus*, *E. maurus*, and *I. cenchoa*. The pools of ticks from the reptiles *T. callirostris* and *K. scorpioides* were not amplified in the conventional PCR assay.

### Phylogenetic analysis

**Figure-3:**
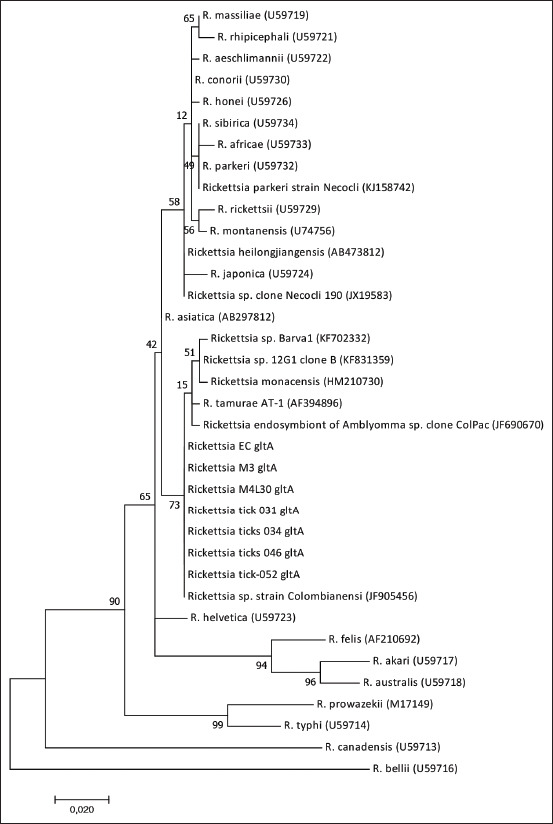
Phylogenetic analysis of the sequences of a 350-bp fragment of the *gltA* gene from Rickettsia colombianensi, amplified from *Amblyomma dissimile*. The tree was built by the maximum likelihood method. The branch supports (Bootstrap) of the evolutionary analysis were made with 1000 replicas in the MEGA X program. The access number of each species of Rickettsia is shown in parentheses.

**Figure-4:**
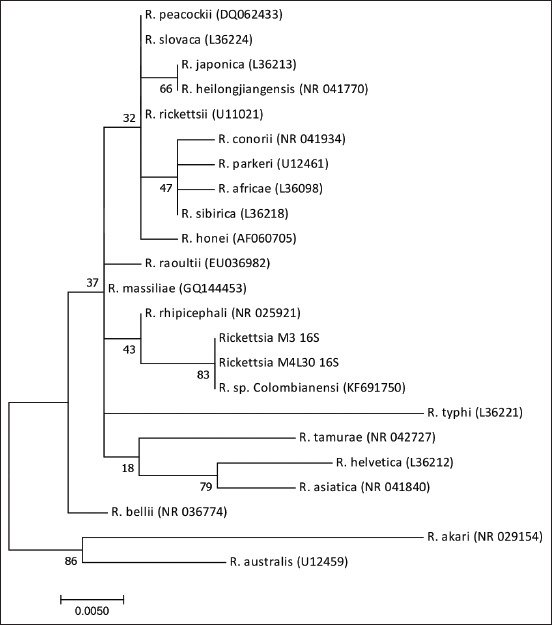
Phylogenetic analysis of the sequences of a 350 bp fragment of the *16S rRNA* gene of the *Candidatus* Rickettsia colombianensi, amplified from *Amblyomma dissimile*. The tree was built by the maximum likelihood method. The branch supports (Bootstrap) of the evolutionary analysis were made with 1000 replicas in the MEGA X program. The access number of each species of Rickettsia is shown in parentheses.

The amplified products of the *gltA* gene revealed nucleotide sequences identical to each other and with 100% similarity to those of *Candidatus* Rickettsia colombianensi (GenBank^®^: KF905456) and *Rickettsia* spp. clone Necocli 190 (JX519583) and 99% similarity to those of *R. tamurae* (AF394896), the closest validated species (Figure-3). The *16S rRNA* gene nucleotide sequences demonstrated 100% identity with *Candidatus* Rickettsia colombianensi (GenBank: KF691750) and 99% identity with *R. rhipicephali* (GenBank: CP003342). Figure-4 shows the phylogenetic analysis of the sequences.

## Discussion

*Candidatus* Rickettsia colombianensi was detected in ticks collected from reptiles in Córdoba, which confirmed that it is a microorganism endemic to the area investigated in this study and possibly endemic throughout Colombia where *A. dissimile* is found. *A. dissimile* was the only tick species found in reptiles analyzed in this study. Similar results have been reported in the previous research conducted in the same geographic area on the green iguana (*I. iguana*) [16] and in other departments (La Guajira, Cesar, and Magdalena), where *A. dissimile* was the only ectoparasite found on the reptiles [22]. In another study, *A. dissimile* was the only tick species found in the toads *Rhinella humboldti* and *Rhinella horribilis* in the Magdalena Department [23]. *A. dissimile* has been described as one of the major ectoparasites of those reptiles kept in captivity and subjected to illegal wildlife trafficking in the Department of Córdoba [24,25].

*A. dissimile* has also been reported in other countries, parasitizing iguanas of the species *Ctenosaura bakeri* and *I. iguana* in Honduras [26] and the snake *B. constrictor* in Costa Rica [27]. This is probably because *A. dissimile* is the most widely distributed tick species in reptiles and amphibians in Central and South America [28]. In contrast to our results, studies conducted in Mexico reported that reptiles were infested either individually or in groups with *Amblyomma mixtum*, *Amblyomma rotundatum*, *Amblyomma sabanerae*, and *Amblyomma scutatum* but not with *A. dissimile* [29]. However, other researchers in Mexico have reported *A. dissimile* infestations on reptiles [30].

To the best of our knowledge, the present study is the first report in Colombia regarding *Candidatus* Rickettsia colombianensi in *A. dissimile* collected from reptiles of the species *T. callirostris*, *T. gambotensis*, *I. cenchoa*, *E. melanotus*, *B. asper*, *E. maurus*, and *K. scorpioides*, with high frequency of detection, between 33% and 100% within the tested groups. In our study, the MIR of *Candidatus* Rickettsia colombianensi in *A. dissimile* ticks was 16%, assuming one positive tick per group. This result is similar to the 15% prevalence in *A. dissimile* in the Departments of Magdalena, Cesar, and La Guajira, Colombia [22]. However, in the previous reports of *Candidatus* Rickettsia colombianensi in *A. dissimile* in Córdoba collected from *I. iguana*, the MIR was 29% [16]. Cotes-Perdomo reported *Candidatus* Rickettsia colombianensi in *A. dissimile* collected from toads (*Rhinella horribilis* and *R. humboldti*) at frequencies of 55% (5/9 adults), 57% (24/41 groups of nymphs), and 88% (28/32 groups of larvae) [23].

*Candidatus* Rickettsia colombianensi has also been reported in *A. dissimile* on the iguanas *C. bakeri* and *I. iguana* and in larvae of *Amblyomma* spp. on the bird species *Geothlypis formosa* in Honduras; However, Novakova *et al*. [26] in the analysis of the sequence of this rickettsia it showed that it had differences in the genetic sequence (0.4%) with respect to Candidatus Rickettsia colombianensi detected in Colombia, for this reason, it was named Candidatus Rickettsia colombianensi genotype Utila. In Brazil, *Rickettsia* that were 100% identical to *Candidatus* Rickettsia colombianensi were detected in a group of ten larval *A. dissimile* collected from toads (*Rhinella marina*) [31].

Ticks of reptiles and amphibians could play an essential role in the transmission of new rickettsial species, with some potential pathogenicity. *Rickettsia* spp. RDa420 is a part of the spotted fever group, described among the ticks *Amblyomma helvolum* and *Amblyomma varanense* collected from the snakes *Python molurus bivittatus*, *Xenochrophis piscator*, *Ptyas korros*, and *Ophiophagus hannah* in Thailand [32]. In Malaysia [33], among the same ticks (*A. helvolum* and *A. varanense*) collected from captured snakes, two new *Rickettsiae*, *Candidatus* Rickettsia sepangensis, similar to *Rickettsia tamurae*, and *Candidatus* Rickettsia johorensis, which is very close to *Rickettsia raoultii*. *Rickettsia bellii* has been described from *A. rotundatum* ticks collected from turtles (*Chelonoidis carbonaria)* that are illegally traded between Israel and the United States [34]. *R. bellii* has also been reported in *A. rotundatum* collected from amphibians (*Rhinella jimi*) from the arid regions of Brazil [35], and in Colombia, it has been described from a group of *A. dissimile* larvae collected from the lizard *Basiliscus basiliscus* and the toad *R. horribilis* in the Department of Magdalena, Colombia [22,23].

The presence of ticks belonging to the genus *Amblyomma* in illegally trafficked reptiles has become a public health concern in the United States and some countries of the European continent, which is because ticks can develop breeding colonies and establish themselves as endemic, affecting the populations of native reptiles and even domestic livestock [11,36].

## Conclusion

The potential danger of illegal trade of reptiles with respect to the spread of diseases has already been described. The finding of *Rickettsia* from the spotted fever group in illegally trafficked reptile ticks would pose a health risk. It is known that these types of *Rickettsiae* are potentially pathogenic for humans, and the trade of live reptiles could be a mechanism responsible for their introduction into countries where they are currently not detected. Our findings suggest that it is important to establish sanitary and veterinary measures to control the trade and holding of captive reptiles and to prevent the potential risk of transmission of *Rickettsia* spp. to humans.

## Authors’ Contributions

JM, LV, and SaM conceived and designed the experiments; JM, LV, SB, and VC collected, classified ticks, and performed the experiments; JM, LV, VC, SM, SaM, and JR analyzed the data, wrote, and revised the manuscript. All authors read and approved the final manuscript.
